# Non-COVID Diseases during the Pandemic: Where Have All Other Emergencies Gone?

**DOI:** 10.3390/medicina56100512

**Published:** 2020-10-01

**Authors:** Veronica Ojetti, Marcello Covino, Mattia Brigida, Carmine Petruzziello, Angela Saviano, Alessio Migneco, Marcello Candelli, Francesco Franceschi

**Affiliations:** 1 Emergency Department—Fondazione Policlinico Universitario A. Gemelli, IRCCS—Largo A. Gemelli, 00168 1 Rome, Italy; macovino@gmail.com (M.C.); alessio.migneco@gmail.com (A.M.); mcandelli@gmail.com (M.C.); francesco.franceschi@unicatt.it (F.F.); 2 Università Cattolica del Sacro Cuore—Largo F. Vito, 00168 1 Rome, Italy; mattiabrigida@hotmail.it (M.B.); saviange@libero.it (A.S.); 3 Ospedale Cristo Re, Emergency Department—Via delle Calasanziane, 00168 25 Rome, Italy; carminepetruzziello@live.it

**Keywords:** COVID-19, Emergency department access, misuse, fever

## Abstract

*Background and objectives:* the emergency department (ED) is frequently identified by patients as a possible solution for all healthcare problems, leading to a high rate of misuse of the ED, possibly causing overcrowding. The coronavirus disease 2019 (COVID-19) pandemic started in China; it then spread throughout Italy, with the first cases confirmed in Lombardy, Italy, in February 2020. This has totally changed the type of patients referred to EDs. The aim of this study was to analyze the reduction of ED admissions at a Second level urban teaching (Fondazione Policlinico Universitario Agostino Gemelli IRCCS) during the COVID-19 pandemic. *Materials and Methods:* in this retrospective observational cross-sectional study, we reviewed and compared clinical records of all the patients consecutively admitted to our ED over a 40-day period (21 February –31 March) in the last three years (2018–2019–2020). Mean age, sex, triage urgency level, day/night admission, main presentation symptom, and final diagnosis, according to different medical specialties, hospitalization, and discharge rate, were analyzed. *Results:* we analyzed 16,281 patient clinical records. The overall reduction in ED admissions in 2020 was 37.6% compared to 2019. In 2020, we observed an increase in triage urgency levels for ED admissions (the main presentation symptom was a fever). We noticed a significant drop in admissions for cardio-thoracic, gastroenterological, urological, otolaryngologic/ophthalmologic, and traumatological diseases. Acute neurological conditions registered only a slight, but significant, reduction. Oncology admissions were stable. Admissions for infectious diseases were 30% in 2020, compared to 5% and 6% in 2018 and 2019, respectively. In 2020, the hospitalization rate increased to 42.9% compared to 27.7%, and 26.4% in previous years. *Conclusions:* the drastic reduction of ED admissions during the pandemic may be associated with fear of the virus, suggesting that patients with serious illnesses did not go to the emergency room. Moreover, there was possible misuse of the ED in the previous year. In particular, worrisome data emerged regarding a drop in cardiology and neurology admissions. Those patients postponed medical attention, possibly with fatal consequences, just for fear of exposure to COVID-19, leading to unnecessary morbidity and mortality.

## 1. Introduction

Severe acute respiratory syndrome coronavirus 2 (SARS-CoV-2) is the causative agent of the recent and current pandemic, coronavirus disease 2019 (COVID-19). Its main manifestations range from fever (89%), cough (68%), fatigue (38%), dyspnea (19%), pharyngodynia (14%), and headaches (14%), to other enteric, hepatic, and neurological symptoms [1,2,3]. Everything seems to have started in late December 2019, in China, with a cluster of patients hospitalized for pneumonia of an unknown etiology. Given the estimates of reproduction for SARS-CoV-2, ranging from 2.24 to 3.58, the scientific community expressed immediate concern about a potential SARS-CoV-2 outbreak [4].

As of 22 January, 2020, a total of 571 cases of COVID-19 were observed in 25 provinces in China. Less than one month later, on 16 February, the number of COVID-19 cases reached 51,857 globally in 25 countries [4,5]. In Italy, the first case was registered on 21 February, 2020 [6]. The first red zone declared in Lombardy was Codogno [7], a town near Milan; soon after, it was extended to the entire region. People were not allowed to enter or exit this area starting from 1 March [8]. The World Health Organization (WHO) declared COVID-19 as a pandemic on 11 March, 2020 [9]. While official news was posted on the Italian Ministry of Health website, a new decree imposed a lockdown, with further restrictions on Italian citizens, until 3 April, with exceptions for health, food, and bank workers. Schools, museums, cinemas, and theaters closed, and all social gathering activities were forbidden. Moreover, social distancing among people was introduced, with obligation to wear facial masks when away from home or inside shops [10]. Subsequently, Italian containment measures became stricter, with a further decree [11] establishing a complete lockdown; however, no restrictions were imposed on individuals who left their homes for health reasons.

Reports from medical facilities around the world seem to suggest that all of these conditions have, seemingly, gone into hiding since the COVID-19 onslaught.

We also observed a significant drop of access to EDs’ during the COVID-19 pandemic.

Therefore, we designed a study aimed at analyzing diagnoses, hospitalization rates, and number of emergency department (ED) admissions at our hospital during the COVID-19 pandemic, from 21 February, 2020 (when the first Italian case was observed) to 31 March, 2020, and compare the results to the same periods in 2018 and 2019.

## 2. Methods

### 2.1. Study Design

This is a single-center, retrospective, cross-sectional observational study, conducted in the ED of an urban tertiary teaching hospital (Fondazione Policlinico Universitario Agostino Gemelli IRCCS).

Fondazione Policlinico Universitario Agostino Gemelli IRCCS is a large university hospital located in Rome, with an annual attendance at the emergency department (ED) of about 75,000 patients (more than 85% being adults).

The study was conducted in accordance with the Declaration of Helsinki.

All patients who accessed our ED signed an informed general consent form for personal data processing.

Approval was received from the Ethical Committee on 20 April, 2020, protocol number 0017055/20.

The database, with de-identified participants used for data retrieval, will be made available, with publication upon written request after approval of a proposal, with a signed data access agreement.

### 2.2. Study Size and Selection Criteria

We reviewed and compared clinical records of all the patients who were consecutively admitted to our ED over a 40-day period (21 February–31 March) in the last three years (2018–2019–2020).

We excluded from the study all patients < 18 years old, and pregnant women who were managed in another ED, in particular pediatric first aid and obstetric first aid.

### 2.3. Study Variables

The following information was extracted from computerized clinical records, GIPSE, the informatic system utilized in our hospital and region.

The system manages the entire patient’s route: from reception, selection, and prioritization of access (triage), to clinical presentation, outcome management, report production, and statistics.

The use of the same procedure on a regional scale supports the standardization of data collection activities and allows for the verification of the quality of care and the standardization of assistance.

Age, sex, triage urgency level, presentation time (day/night), main presentation symptom, final diagnosis according to different medical specialties (based on the International Classification of Diseases, 9th revision, Clinical Modification (ICD-9-CM)) [12], hospitalization, and discharge rate, were analyzed.

The numeric triage:

- Code 1: indicates maximum emergency; the patients to whom it is assigned have immediate access to the treatment areas.
- Code 2: indicates urgency, and gives access to emergency room care within 15 min of being assigned.
- Code 3: a deferrable urgency that does not require immediate intervention.
- Code 4: when the urgency is less.
- Code 5: this value indicates a situation of no urgency.

In a subgroup analysis, we considered three different age groups: “adults”, aged between 18 and 64 years; “elderly” aged between 65 and 84 years; and “oldest old” aged over 85 years.

### 2.4. Study Endpoint

The primary endpoint was to evaluate the number of total admissions, diagnoses, and hospitalization rates during the analyzed period in 2020, and compare these data to the previous two years (2018–2019).

The secondary endpoint was to show the daily variation in trends of ED visits and trends for ED admissions for fever in the analyzed time interval, for 2018, 2019, and 2020.

### 2.5. Statistical Analysis

Categorical variables were statistically compared with the chi-square test. Continuous variables were compared with the *t*-test. Categorical variables are presented as numbers and percentages, and continuous variables are presented as mean ± standard deviation. A *p*-value of 0.05 or less was considered significant. All data were analyzed with SPSS v25^®^ (IBM, Armonk, NY, USA).

There were no missing data to be addressed.

## 3. Results

We retrospectively analyzed all of the 16,281 patients.

### 3.1. Total Admissions

General characteristics of the patients in the three periods are summarized in Table 1.

**Figure 1:**
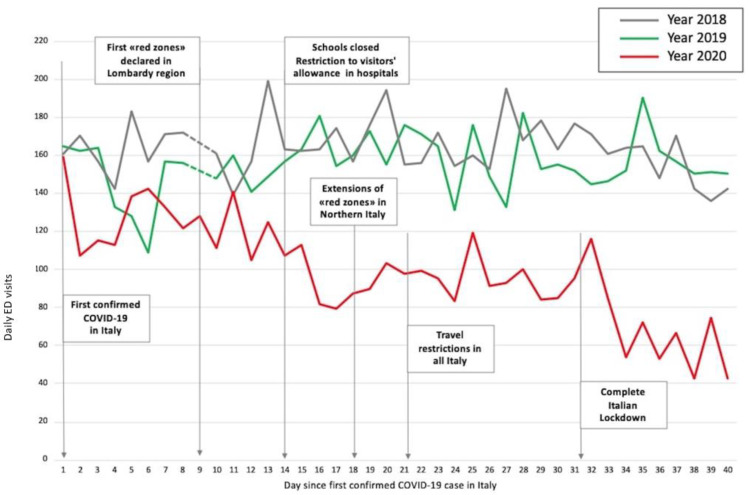
Variation in trends of emergency department (ED) visits.

Total number of ED admissions were constant during the years 2018 and 2019 (6001 and 6329, respectively) while a drastic decrease was observed in the same period of 2020 (3951). The overall reduction in ED admissions in 2020 was 34.2% compared to 2018, and 37.6% compared to 2019. (Figure 1).

We did not find any significant differences (*p* = 0.303) in the average age of the patients analyzed in different years: mean age was 57.7 ± 20.3 years in 2018, 57.6 ± 20.5 in 2019, and 58.3 ± 19.1 in 2020.

In 2018, 58% of patients were adult, 31% were elderly, and only 11% over 85 years old. The same breakdown of patients by age group was highlighted in 2019 and 2020.

In 2020, compared to the previous two years, we observed an increase of triage urgency level at ED admission (*p* < 0.001). In particular, there was a reduction of non-urgent conditions, whilst we registered an increase of moderate urgency (2018: 39.0% vs. 2019: 39.5% vs. 2020: 46.2%) and emergency (2018: 6.4% vs. 2019: 6.2% vs. 7.8%).

Regarding presentation time, we found a slight reduction (*p* < 0.05) of night admissions in 2020.

**Figure 2:**
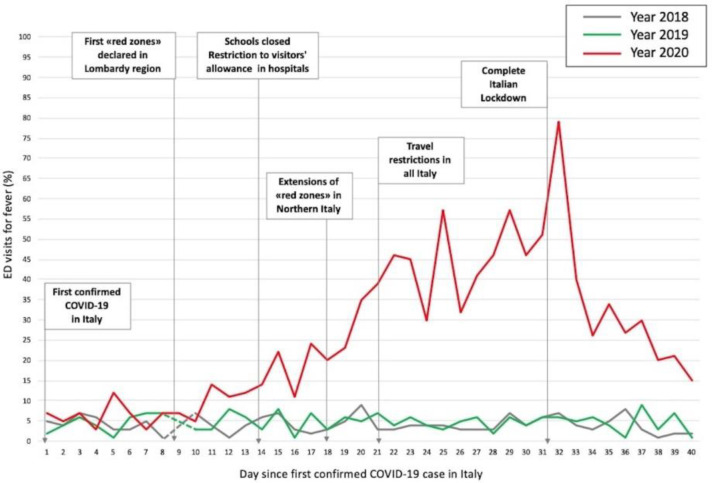
Trends for ED admissions for fever.

Main ED presentation symptoms were fever, pain, trauma, and dyspnea in 2018 and 2019, whereas in 2020 most of the patients presented with fever (Figure 2).

### 3.2. Admissions Divided by Specialties

ED visit outcomes are analyzed in Table 2.

We noticed a significant drop in admission for cardio-thoracic, gastroenterological, urological, otolaryngologic/ophthalmologic, and traumatological diseases. Acute neurological conditions registered a slight, but significant, reduction. Interestingly, oncology admissions were stable.

On the other hand, a significant increase in admissions for infectious causes was registered in 2020 (*p* < 0.001). The confirmed case of COVID-19 were 516 out of 3951 (13%). The other 515 patients who came with fever obtained a diagnosis of “fever for other causes”; this could be due to the suggestions from our health system to visit the ED for persistent fever.

### 3.3. Hospitalization Rate

Concerning the overall hospitalization rate in the three years taken into consideration (summarized in Table 2), we observed how 27.7% of patients were hospitalized in 2018 and 26.4% in 2019; conversely, hospitalization rate increased to 42.9% in 2020; thus, suggesting a greater severity of patients who were referred to the ED during the COVID-19 pandemic.

The percentage of deceased patients in the ED was stable in the three years. In 2020, we registered a slight reduction of patients spontaneously leaving the ED (i.e., self-discharging).

### 3.4. Trends in ED Visits

Daily trends in ED visits (plotted in Figure 1) were severely affected by government dispositions to contrast the pandemic spread.

After the first case diagnosed in Italy on 21 February, we observed a progressive reduction in ED total visits, which was lower compared to 2018 and 2019.

With the first measures affecting all of Italy, we observed a further slight drop in ED admissions, which had been stable until the complete lockdown decision occurred on 21 March. Since then, total ED visits were mainly dominated by COVID-19 cases.

Furthermore, we described trends in fever presentation of the admitted patients (Figure 2). In 2018 and 2019, the overall fever-related visits were stable, being about 6% of total admissions. The COVID-19 pandemic produced significant changes in this regard; after the very first days of March, the number of fever-related visits steadily increased, reaching a peak on 22 March, just after the Italian lockdown was declared.

## 4. Discussion

The main finding of this study is the demonstration of a significant drop in the number of patients accessing the ED for non-serious conditions and low priority codes.

It is well known that the conditions for which patient’s access the ED include myocardial infarction, strokes, respiratory diseases, injuries, road traffic accidents.

Moreover, it is very common to observe in our hospital, during the first months of the year, ED overcrowding, partially due to the winter virus season and, in part, to inappropriateness of access for non-urgent visits [13,14]. On this subject, some authors suggested that around 20–30% of ED visits may be defined as inappropriate, probably due to some critical issues inside national health systems, such as lack of primary care centers and low collaboration with general practitioners (GPs) [15].

Concerning main characteristics of the patients, in our study, mean age of admitted patients remained unchanged in 2018–2019 and 2020. However, when looking at the male/female ratio, we noticed a shift from a female prevalence in 2018 and 2019 to a male predominance.

This finding may be linked, also, to a slightly higher prevalence of male patients affected by COVID-19, especially those with specific comorbidities, such as blood hypertension, diabetes, cardiovascular disease, or chronic lung disease [16,17].

Concerning triage priority codes, we observed a significant increase in patients with code 2 (moderate urgency) previously defined as yellow code and code 1 (emergency), previously defined as red code, reflecting a high number of patients who accessed the ED with significant respiratory problems and consequent vital parameters alteration (i.e., oxygen saturation), which are taken into account to determine triage code [18]. Interestingly, a recent paper published by a group in Lodi, an Italian city with a very high COVID-19 prevalence, reported a complete disappearance of patients with priority codes 4 and 5 (previously defined as green and white) [19].

This could be due to the role of territorial medicine, which perhaps favored the prevalence of only codes 1 and 2 in the hospital, effectively managing the minor urgency.

As illustrated in Table 1 and Figure 2, the pandemic also had a strong impact concerning prevalence of symptom presentation at ED admission, when compared to the previous two years. In particular, a progressive increase of the symptom “fever” was observed until complete lockdown occurred on 22 March, after which fever-related admissions dropped.

Another interesting issue that emerged from our analysis was the prevalence of patients who came to the ED for pain symptom. While, in 2018 about 16.5% of total ED visits were recalled to pain, and a similar percentage was observed in 2019, during the 2020 COVID-19 pandemic outbreak, prevalence of these patients dropped to 11.6%, suggesting that many patients preferred an in-home management of pain rather than going to the ED (*p* < 0.001).

Collaterally, a reduced admission for trauma has also been observed, while a reasonable explanation may be found in the protective effect of lockdown on minor traumas. However, admissions for traumas remained basically unchanged.

One of the most important considerations coming out from our analysis regards admission for cardiovascular diseases during the pandemic phase, which reported a decrease of about 46% compared to 2019 data. Several reports showed a significant reduction of accesses in the ED for cardiovascular diseases; in the Sicily region, there was a drop in 50% of accesses [20], while in Milan, the COVID-19 pandemic has tripled acute coronary syndrome deaths and reduced life-saving procedures by 40% [21]. A recent paper published in *The Lancet* suggests that during the COVID-19 pandemic, there was a reduction of admission rates for (and management of) acute coronary syndromes in England [22]. This data was also noted in our studies. Our concern is that those patients postponed medical attention, possibly with fatal consequences, just for fear of exposure to COVID-19 [23].

Others suggested there was a significant drop in cardiac events linked to unavailability of alcohol beverages and tobacco products, the drop in environmental pollution, and reduction of job-related and travel-related stress, with better or controlled sleeping hours [24].

Another similarly worrisome aspect concerns the neurological admission drops that we observed during the high prevalence pandemic phase, compared to the same periods in 2018 and 2019. This is consistent with data coming from an inquiry conducted in 81 centers belonging to the neurovascular unit network for pharmacological and/or mechanical revascularization in Italy. Therefore, further evaluation of this phenomenon will probably be required once we return to normal conditions [25].

On the other hand, not all admission reductions were equally concerning; we are less worried about the 51% reduction of access for Gatrointestinal (GI) conditions, considering that many of these are chronic and not acute diseases, which could be easily managed by the GP or GI specialists. Similarly, urological conditions (e.g., renal colic) and infectious otolaryngologic diseases could, as well, be handled by specialists, prescribing home appropriate treatments, possibly with a telemedicine approach [26].

No variation about patients accessing the ED for malignancies was observed, mostly due to the opening of chemotherapy services, also, during the high prevalence pandemic phase.

On the contrary, a significant increase of access for infectious causes, which also included COVID-19 infection, was observed, as largely expected. The most relevant finding was the increased prevalence of patients with those characteristics, from 5.5% of 2018 to 30% of the pandemic period of 2020. Indeed, the Italian COVID-19 contagion curve was describing an exponential trend during the time interval considered for our study [27].

Concerning hospitalization, the absolute number of hospitalized patients is roughly the same in the high prevalence pandemic phase compared to the same period of the last 2 years. There was only an increase in terms of hospitalization rate in 2020, but this was mostly due to a drop in patients with low priority codes, and it may be then considered as a pure artifact. The main problem was found in the characteristics of hospitalized patients, mostly affected by severe respiratory problems, forcing our hospital to review and modify its usual organization, by increasing intensive and semi-intensive beds, and improving the number of ventilators. This was perfectly in line with what was observed in other hospitals, in which structural and logistical strategies were implemented in order to prepare to receive hundreds of patients with different respiratory requirements, keeping clean emergency access lines, restoring surgical interventions, and deferring urgent ordinary activities [19,28].

Finally, our subgroup analysis on different age groups revealed that, in the study period, the overall distribution of all-cause admissions did not change from previous years. However, focusing on distribution of infectious disease admissions, we observed a 227.6% increase in adults (N2019 = 199 vs. N2020 = 652), a 174.5% increase in elderly (N2019 = 153 vs. N2020 = 420), and a 111.1% increase in oldest old (N2019 = 54 vs. N2020 = 114); thus, suggesting a more severe impact of COVID-19 on the admission of patients belonging to the adult group, as observed by other authors [29,30,31].

A limitation of our study is probably its monocentric nature, and that Fondazione Policlinico Universitario Agostino Gemelli was one of the referral centers for COVID-19 in Rome. We do not know if these patients accessed other EDs, or if they stayed home with an ongoing disease process.

A potential confounder in our study could be the concomitant *influenza* pandemic of the winter season.

To date, there are no other studies reporting trends in ED admissions during the pandemic for such a large adult population. However, our data seem to be generalizable in light of the many TV news reports, suggesting decreasing trends throughout all EDs in Italy.

## 5. Conclusions

In conclusion, our data highlight that, during the analyzed time interval, ED admissions were mostly related to the COVID-19 disease or complications. The main presentation symptoms registered for overall admissions were fever and dyspnea, and the most frequent ICD-9 diagnostic group was “Infectious Diseases”.

The drastic reduction of ED admissions during the pandemic may be associated with fear of the virus, suggesting that patients with serious illnesses did not come to the emergency room and, on the other hand, there was possible misuse of the ED in the previous year. According to national emergency departement overcrowding study (NEDOCS) indices, measuring ED overcrowding, the drop was close to 70%. Even without using complex algorithms, it was very easy to observe how few patients were located inside the ED and waiting rooms.

Specific government information programming on COVID-19, which suggested patients not access the ED, but call their GP, or dedicate numbers, together with the fear of coming in contact with infected patients inside EDs, was surely responsible for the lower access rate during this pandemic phase. This modality needs to be implemented even after the pandemic phase, in order to increase the role of GPs in the management of patients with non-evolutive conditions, which may also be managed outside the ED.

Reduction of ED access, however, does not have to be related to time-related diseases, such as acute coronary syndrome and stroke, as it may dramatically increase overall mortality. Patients need to be appropriately solicited not to pursue this modality.

## Figures and Tables

**Table 1:** Demographics, age group distribution, triage urgency levels, presentation time, and main presentation symptom from 21 February to 31 March in 2018, 2019, and 2020.

Variable	Year 2018 (*n* = 6001)	Year 2019 (*n* = 6329)	Year 2020 (*n* = 3951)	*p* Value
Sex	2887/3114	3127/3202	2101/1850	<0.001
(Male%/Female%)	(48.1%/51.9%)	(49.4%/50.6%)	(53.2%/46.8%)	
Age (median (IQ range))	58.0 (42.0–75.0)	58.0 (42.0–75.0)	58.0 (44.0–75.0)	0.303
Age group				0.126
- 18–64 years	3525 (58.7%)	3748 (59.2%)	2343 (59.3%)	
- 65–85 years	1914 (31.9%)	1960 (31.0%)	1277 (32.3%)	
- ≥ 85 years	562 (9.4%)	621 (9.8%)	331 (8.4%)	
Triage				<0.001
1 Emergency	384 (6.4%)	392 (6.2%)	308 (7.8%)	
2 Moderate Urgency	2340 (39.0%)	2500 (39.5%)	1825 (46.2%)	
3 Non Urgent	3163 (52.7%)	3380 (53.4%)	1755 (44.4%)	
4 Ambulatory	114 (1.9%)	57 (0.9%)	63 (1.6%)	
Presentation Time				0.028
Day (8 a.m.–7 p.m.)	4279 (71.3%)	4538 (71.7%)	2912 (73.7%)	
Night (8 p.m.–7 a.m.)	1722 (28.7%)	1791 (28.3%)	1039 (26.3%)	
Main presentation symptom				
Pain	990 (16.5%)	987 (15.6%)	458 (11.6%)	<0.001
Fever	162 (2.7%)	177 (2.8%)	1031 (26.1%)	<0.001
Dyspnea	318 (5.3%)	303 (4.8%)	300 (7.6%)	<0.001
Trauma	846 (14.1%)	981 (15.5%)	336 (8.5%)	<0.001

**Table 2:** ED admissions distributed according to different diagnostic codes (from the International Classification of Diseases, 9th revision, Clinical Modification, ICD-9) and ED visit outcome.

Variable	Year 2018 (*n* = 6001)	Year 2019 (*n* = 6329)	Year 2020 (*n* = 3951)	*p* Value
Diagnosis group *				
Cardio-thoracic	1152 (19.2%)	1189 (18.8%)	644 (16.3%)	0.001
Neurological	432 (7.2%)	417 (6.6%)	233 (5.9%)	0.027
Gastrointestinal	768 (12.8%)	747 (11.8%)	363 (9.2%)	<0.001
Urogenital	270 (4.5%)	335 (5.3%)	134 (3.4%)	<0.001
Malignancy	240 (4.0%)	209 (3.3%)	142 (3.6%)	0.130
Traumatology	810 (13.5%)	816 (12.9%)	332 (8.4%)	<0.001
Otolaryngology/Ophthalmology	192 (3.2%)	228 (3.6%)	91 (2.3%)	0.001
Infectious disease	330 (5.5%)	405 (6.4%)	1185 (30.0%)	<0.001
ED visit outcome				
Self-discharging	414 (6.9%)	430 (6.8%)	213 (5.4%)	0.005
Deceased in ED	36 (0.6%)	32 (0.5%)	12 (0.3%)	0.058
Admitted	1662 (27.7%)	1671 (26.4%)	1694 (42.9%)	<0.001

* Minor diagnostic groups from the ICD-9 classification were not included in the analysis.
